# Evaluation of the risk factors contributing to the African swine fever occurrence in Sardinia, Italy

**DOI:** 10.3389/fmicb.2015.00314

**Published:** 2015-04-14

**Authors:** Beatriz Martínez-López, Andres M. Perez, Francesco Feliziani, Sandro Rolesu, Lina Mur, José M. Sánchez-Vizcaíno

**Affiliations:** ^1^Center for Animal Disease Modeling and Surveillance, Department of Medicine & Epidemiology, School of Veterinary Medicine, University of CaliforniaDavis, Davis, CA, USA; ^2^Department of Veterinary Population Medicine, College of Veterinary Medicine University of MinnesotaSaint Paul, MN, USA; ^3^Istituto Zooprofilattico Sperimentale dell’Umbria e delle MarchePerugia, Italy; ^4^Osservatorio Epidemiologico Veterinario Regionale, Istituto Zooprofilattico Sperimentale della SardegnaCagliari, Italy; ^5^Animal Health Department and Centro de Vigilancia Sanitaria Veterinaria, Veterinary School, Complutense University of MadridMadrid, Spain

**Keywords:** Bayesian model, risk-based surveillance, eradication program, spatial epidemiology, backyard pigs

## Abstract

This study assesses the relation between hypothesized risk factors and African swine fever virus (ASFV) distribution in Sardinia (Italy) after the beginning of the eradication program in 1993, using a Bayesian multivariable logistic regression mixed model. Results indicate that the probability of ASFV occurrence in Sardinia was associated to particular socio-cultural, productive and economical factors found in the region, particularly to large number of confined (i.e., closed) farms (most of them backyard), high road density, high mean altitude, large number of open fattening farms, and large number of pigs per commune. Conversely, large proportion of open farms with at least one census and large proportion of open farms per commune, were found to be protective factors for ASFV. Results suggest that basic preventive and control strategies, such as yearly census or registration of the pigs per farm and better control of the public lands where pigs are usually raised, together with endanced effords of outreach and communication with pig producers should help in the success of the eradication program for ASF in the Island. Methods and results presented here will inform decision making to better control and eradicate ASF in Sardinia and in all those areas with similar management and epidemiological conditions.

## Introduction

African swine fever (ASF) is caused by the infection with a complex and large DNA virus (ASFV) of the *Asfarviridae* family (Salas, 1999; Dixon et al., 2005). ASF is a haemorrhagic disease of pigs in which clinical signs depend on the virulence of the ASFV isolate, dose, and route of infection and host (domestic or wild pig). Clinical presentation may vary from a hyperacute form, with almost 100% mortality after 4–7 days post-infection, to a chronic or an asymptomatic form in which most of animals may survive and become carriers (Sánchez-Vizcaíno et al., 2012).

African swine fever is, arguably, one of the most difficult to control and economically devastating viral diseases of pigs. Difficulty to control ASF and the economic impact of the disease are consequence of a multiplicity of factors, including the role played by soft ticks (*Ornithodoros* genus) on disease transmission, lack of an effective vaccine to prevent ASF infection, long persistence of the virus in the environment and in pig products, presence of asymptomatic and carrier animals, and severe restrictions to the international trade of pigs and their products imposed to regions in which the disease is known or suspected to be present (Sánchez-Vizcaíno et al., 2012).

After the first description of the disease by Montgomery (1921) in Africa, the disease rapidly spread during 1960s and 1970s, from Sub-Saharan African countries into Europe, and Central and South America. The ASFV also recently spread into Georgia, Armenia, Azerbaijan, and the Russian Federation, which are countries that were believed to be ASF-free prior to 2007 (Food and Agriculture Organization [FAO], 2009). ASF has been eradicated from the Americas and Western Europe, with the exception of the Mediterranean island of Sardinia, where the disease has been endemic since 1978. Although a rigorous European Union (EU)-supported ASFV eradication program has been in place in Sardinia since 1993 (EU Official Bulletin no. L 116, 1990), ASFV outbreaks are still reported on an annual basis in the island or even have increased in the last years in some areas. The ASF eradication program for Sardinia has been continuously evolving, but the basics are found on the Council Decision of April 25, 1990 (EU Official Bulletin no. L 116, 1990) which comprises measures for (i) a rapid elimination of ASF outbreaks (i.e., immediate killing, destruction, disinfection, and compensation of owners after an outbreak, etc.), (ii) surveillance and protection of pig farms (i.e., serological testing of pigs in high risk areas, epidemiological outbreaks investigations, pre-movement tests, sampling of wild pigs, etc.), (iii) identification of pigs and pig farms, and (iv) construction of facilities for sanitary control. Notice that the ASF eradication program in Sardinia is similar to other ASF eradication programs conducted, for example, in countries such as Spain or Portugal, although in those two countries ASF has been successfully eradicated.

Presence of ASF-infected territories in Sardinia seems not to pose a major threat to the pig industry of EU-free regions, given that only one outbreak was caused in northern Italy in 1983, by illegal introduction of pork from Sardinia (Mannelli et al., 1998). However, its presence inflicts severe economic losses, not only for EU and Italy, because of the funds invested in ASF control and eradication programs, but also for local producers due to the trade restrictrions and the depreciation of their pig products. In studies conducted in the 1990s it has been hypothesized that the ASF eradication program in Sardinia has failed because of a variety of risk factors that characterize the traditional pig management practices of farmers in the island, including, for example, extensive premises with nil or insufficient biosecurity measures in place, illegal trade and production of pigs and pig, presence of wild boars, and use of waste to feed pigs (Wilkinson, 1984; Mannelli et al., 1997, 1998). Conversely, there is no evidence that soft ticks play any role in the maintenance or spread of ASFV-infection in Sardinia (Ruiu et al., 1989). Noteworthy, no peer-review recent studies have been published assessing association between ASFV infection and epidemiological factors that could influence disease occurrence in Sardinia after more than 15 years (1993–2011) of ASFV eradication program. Such knowledge will contribute to drive the allocation of human and financial resources to support the eradication of ASF in the only region of the EU that remains endemic for the disease. Moreover, the pig production and epidemiological situation in Sardinia may be comparable to those observed in the regions that have been recently infected in the Caucasus region and Russian Federation, and for that reason, this study may contribute to better prevent and control the disease also in those areas with similar management and epidemiological conditions (Food and Agriculture Organization [FAO], 2009).

In the study here, Bayesian modeling was used to explore the nature and extent of the association between ASF occurrence in Sardinia from 1993 to 2009, and hypothesized risk factors for the disease. Results will provide quantitative knowledge on the spatial distribution of ASF in Sardinia and on factors that have influenced occurrence of ASF in Sardinia since 1993. Such knowledge will help to improve the effectiveness of ASF-eradication program in Sardinia and in other ASF-infected territories.

## Materials and Methods

### Unit of Analysis and Data Collection

**FIGURE 1:**
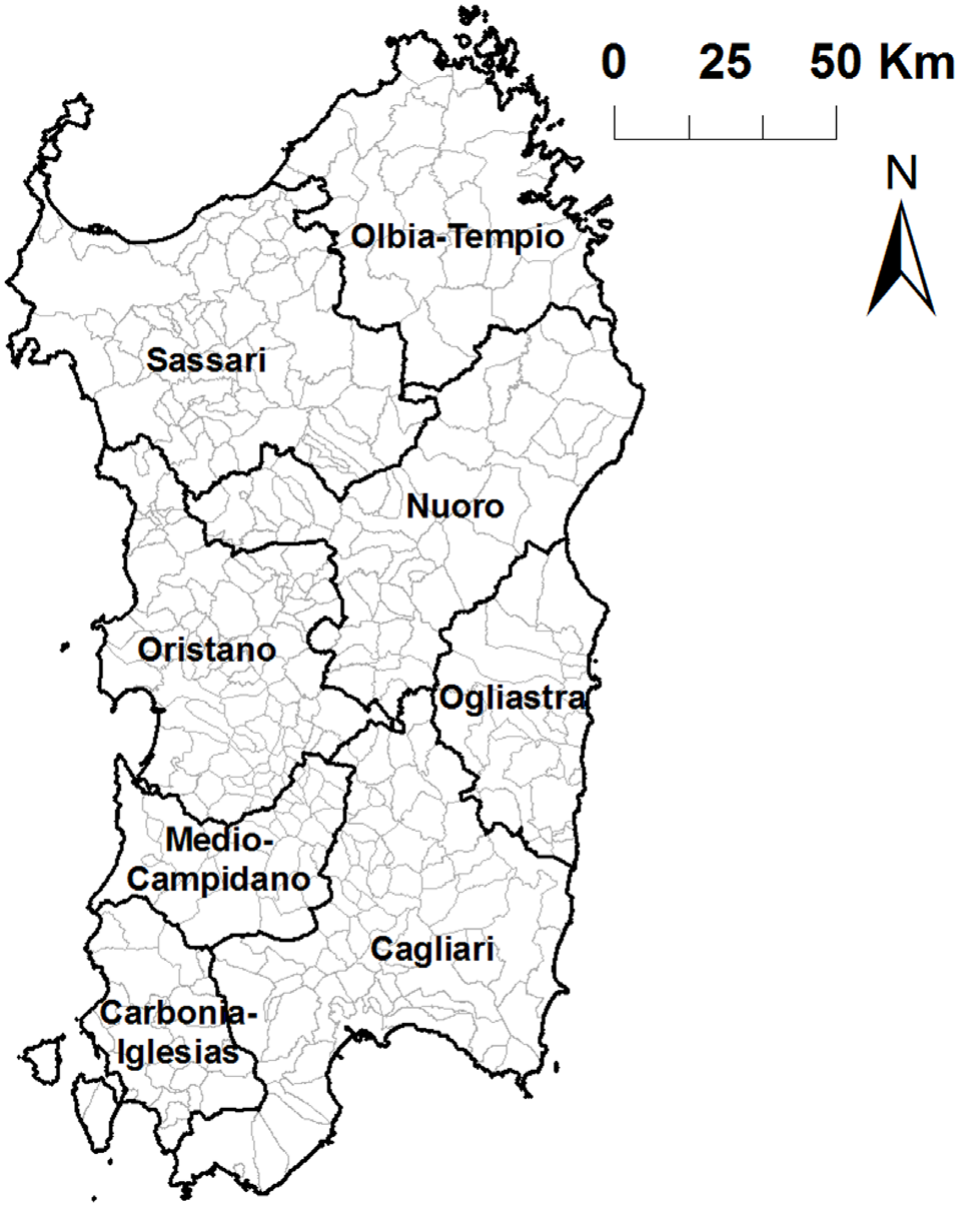
Provinces (dark lines) and commune (narrow gray lines) of Sardinia region of Italy

The unit of analysis here was the smallest administrative division in Sardinia for which ASFV outbreaks were reported, which is referred to as commune. The commune is the basic administrative unit of Italy and it may be considered the third level of the country’s administrative organization, where the first and second administrative aggregations are the region and the province, respectively. Sardinia, which is the second largest island in the Mediterranean Sea, is one of the 20 regions of Italy and it is divided in eight provinces and 376 communes. The mean area of a commune in Sardinia is 64 Km^2^ (**Figure 1**).

An extensive review of the existing data on the distribution of potential risk factors for ASFV outbreak occurrence in Sardinia was conducted to ensure the completeness and reliability of the study. Firstly, potential risk factors for ASFV occurrence in Sardinia were identified searching in “MEDLINE” and “ISI-Web of Knowledge” databases by using a combination of English and Italian keywords such as of “African swine fever,” “peste suina africana,” “risk,” “Sardinia,” “Italy,” “epidemiology,” “cinghiale,” etc. As a result of this literature review, 153 references were selected; and the most updated and reliable information data on the identified risk factors were gathered per commune from national and international sources (**Table 1**). Moreover, selection of risk factors was validated during an expert opinion elicitation (including the administration of a paper questionnaire) conducted on May 20th, 2010 in Oristano, Sardinia, in which more than 25 local veterinary authorities involved in the current ASF control and eradication program were asked to identify the main risk factors for ASF occurrence in the island.

**Table 1:** List of variables used in the Bayesian model for African swine fever in Sardinia.

Variable	Mean (min, max)*	Standardized	Binomial (Codification 0/1 using median)	Source
(1) The number of open fattening farms	4 (0, 61)	YES	na	A
(2) The number of open farms for self-consumption	17 (0, 146)	YES	YES	A
(3) The number of open farrowing farms	20 (0, 366)	YES	YES	A
(4) The number of open farms with no specification of the production type.	3 (0, 46)	YES	na	A
(5) The number of confined/closed farms	23 (0, 153)	YES	YES	A
(6) The total number of open farms	44 (0, 421)	YES	YES	A
(7) The number of sows	143 (0, 864)	na	YES	A
(8) The number of boars	37 (0, 511)	YES	YES	A
(9) The total number of pigs	226 (0, 912)	YES	YES	A
(10) The average number of farms with incoming or outgoing pig movements per month	1 (0, 28)	YES	YES	A
(11) The average number of pigs introduced into farms per month	10 (0, 433)	YES	YES	A
(12) The average number of pigs leaving farms per month	77 (0, 5886)	YES	YES	A
(13) The average number of pigs sent to the slaughterhouse per month	73 (0, 5884)	YES	YES	A
(14) The average number of new born pigs per month	5 (0, 273)	YES	na	A
(15) The area of the Commune (Km^2^)	64 (3, 545)	YES	na	B
(16) The mean altitude (meters)	663 (305, 1991)	YES	YES	B
(17) The density of water areas	0.32 (0, 6.09)	YES	YES	B
(18) The density of roads	0.27 (0, 0.83)	YES	YES	B
(19) The proportion of areas suitable for wild boar presence (\%)	50 (0, 99)	YES	YES	C
(20) The proportion of open farms with at least one census (i.e., farms declaring the number of pigs on farm at least once per year; \%)	80 (2, 100)	YES	YES	A
(21) The number of male people	2176 (50, 72684)	YES	YES	D
(22) The number of female people	2263 (44, 84267)	YES	YES	D
(23) The number of people (human population)	4439 (94, 156951)	YES	YES	D
(24) The mean farm size (number of pigs/number of farms)	4 (0, 29)	YES	YES	A
(25) Pig density (number of pigs/Km^2^)	5 (0, 39)	YES	YES	A
(26) Open farm density (number of farms/Km^2^)	1 (0, 5)	YES	YES	A
(27) Proportion of open farms (number of open farms/total farms) (\%)	69 (0, 100)	YES	YES	A
(28) Density of grazing areas in the commune (Km^2^)	1432 (0, 27806)	YES	YES	B
(29) Proportion of area used as grazing areas in the commune (density of grazing areas in the commune Km^2^/ total area of the commune in Km^2^) (\%)	20 (0, 75)	YES	YES	B

*descriptive statistics of the original (i.e., not standardized) variable.

*na = Not applicable*

*A = Anagrafe Nazionale Zootecnica – Statistiche (2011).*

*B = Servizio Informativo e Cartografico Regionale, Regione Sardegna (2011).*

*C = Corine Land Use (2000) and Rolesu et al. (2007).*

*D = Istituto Nazionale di Statistica [ISTAT] (2011).*

For each commune, information on the number of ASFV outbreaks, pig and human demographics, pig movements and environmental and geographic factors was collected. Specifically, the number of ASFV outbreaks reported per commune from January 1993 to March 2009 was provided by the Istituto Zooprofilattico Sperimentale della Sardegna and by the Istituto Zooprofilattico Sperimentale dell’Umbria e delle Marche. Each farm confirmed as ASFV infected was considered an ASFV outbreak. For each ASF outbreak information regarding the unique identifier and type of farm, farm location, number of pigs present on farm, number of pigs with ASF compatible clinical symptoms, number of death pigs, the day of ASFV detection on farm, day of ASFV confirmation by laboratory, and day of farm depopulation were available.

Data regarding demographics, production type and pig movements per commune in 2009 were obtained from the Italian National Register (Anagrafe Nazionale Zootecnica – Statistiche, 2011). Briefly, just consider that pig production in Sardinia is characterized by traditional pig farming systems, with none or low biosecurity measures in place, either in closed/confined farms (65.4%) or in open/extensive areas (34.6%). Most (77%) of pig farms in Sardinia have less than 25 pigs on farm and more than 41% of the total number of farms are classified as farms for familiar consumption.

The Corine Land Use (2000), from the European Environmental Agency, was used to estimate the areas suitable for the presence/absence of wild boar populations. All agro-forestry areas, bushes and cork oak plantations (CLC_codes: 311–313 and 321–324) were selected as areas with potential presence of wild boar populations, following Rolesu et al. (2007). Other variables such as size of the human population, density of roads, or water areas (i.e., rivers, lakes, or inland waterways), density of grazing lands, and mean altitude were used as proxies for the estimation of the potential feeding of pigs with waste food, legal, or illegal pig trade and the presence of illegal free range pigs, respectively. For example, mean altitude per commune was computed based on detailed (20 m^2^) raster maps provided by the Istituto Zooprofilattico Sperimentale della Sardegna using the zonal statistic function on ArcMap 9.2 (ESRI^®^;). Size of human population per commune in 2009 was obtained from the Istituto Nazionale di Statistica [ISTAT] (2011). Roads, water areas and mean altitude were obtained from the Regional Geographical Service (Servizio Informativo e Cartografico Regionale, Regione Sardegna, 2011). All available information was organized and stored in a Microsoft^®^; Office Access 2007 database.

Although sources and data used for this study were, to the best of the authors’ knowledge, the most complete and reliable information available, information regarding pig farms and pig population is imperfect, as it has been highlighted in the Italian National Register (Anagrafe Nazionale Zootecnica – Statistiche, 2011). Moreover, it should be noticed that although data regarding ASF outbreaks corresponded to 1993–2009, covariate information available to us was collected only in 2009. Because a wide year-to-year fluctuations in the traditional pig demographics, movements and husbandry system in Sardinia was considered unlikely as suggested by Mannelli et al. (1998), the assumption that information in 2009 was representative of the entire period was considered an acceptable simplification of our model.

### Model Approach

A Bayesian multivariable logistic regression mixed model was used to quantify the strength of the association between ASFV-infected communes and epidemiological factors hypothesized to influence the presence of ASF in Sardinia. The response variable was whether or not the commune reported ASF outbreaks from 1993 to 2009 (yes, no). Candidate variables to fit the prediction were each of the 29 epidemiological factors for which information was collected and their second-order interactions (**Table 1**). Candidate variables were alternative modeled as standardized continuous variables or categorized as binomial variables (above, below the median). After identifying a strong spatial structure in the data using a Bernoulli scan statistic model (SaTScan v9.1.1.), we decided to include in the model spatially structured (*S*_i_) and unstructured (*U*_i_) random effects to account for unmeasured factors that had some spatial structure and that were randomly distributed, respectively. Non-informative Normal priors of the form (0, 4) were used for the intercept and the regression coefficients. A non-informative normal prior with mean = 0 and precision = dgamma (0.5, 0.001) was assumed for *U*_i_. An intrinsic Gaussian autoregressive (CAR) structure was used to model Si, where the prior distribution of each *S*_i_ was conditional on the value of the response variable in an adjacent commune (i.e., those communes sharing common boundaries). The precision for *S*_i_ was assumed to be dgamma (0.5, 0.001).

The model was fitted using WinBUGS v.1.4. (Spiegelhalter et al., 2002), with 20,000 iterations, after the first 1,000 samples were discarded as burn-in. Model was built by using a manual forward selection process (i.e., introducing one variable at a time). The model with the lowest deviance information criterion (DIC) value was considered the one that best fitted the data (Spiegelhalter et al., 2002). R2WinBUGS package (Sturtz et al., 2005) in R-language software (R Development core team, 2011) was used to run the models in WinBUGS 1.4. The final model was assessed using autocorrelation plots to visually verify absence of autocorrelation in the predictions. Convergence of the model was also checked by using Gelman–Rubin Plots. The standard deviations (SD) of *S*_i_ and *U*_i_ were used to compare the variability of structured and unstructured random effects, as a proxy to estimate whether epidemiological factors not included in the model were spatially correlated or not. Maps were generated using ArcMap 9.2 (ESRI^^®^;^).

## Results

**FIGURE 2:**
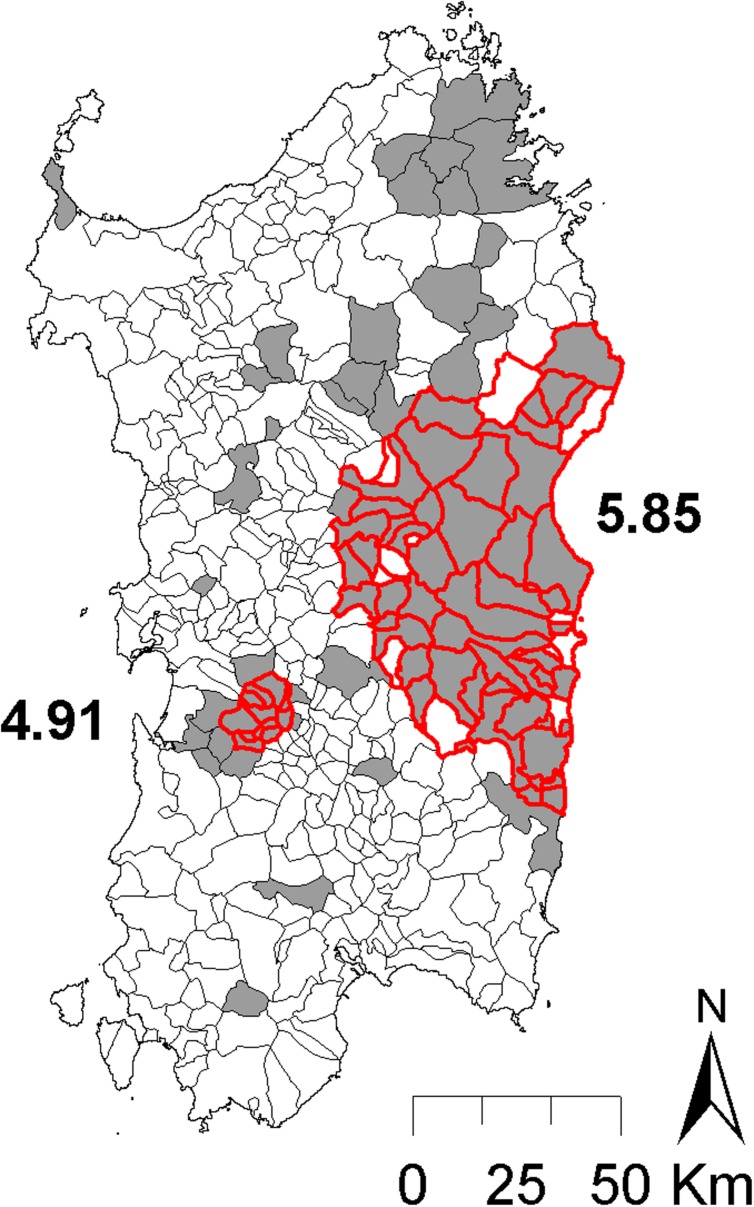
Spatial distribution of African swine fever virus (ASFV) positive (gray filled) communes in Sardinia from 1993 to 2009 with detail of the location (red border) and relative risk values (numbers) for the two significant spatial clusters obtained in a Bernoulli spatial scan statistic model

**FIGURE 3:**
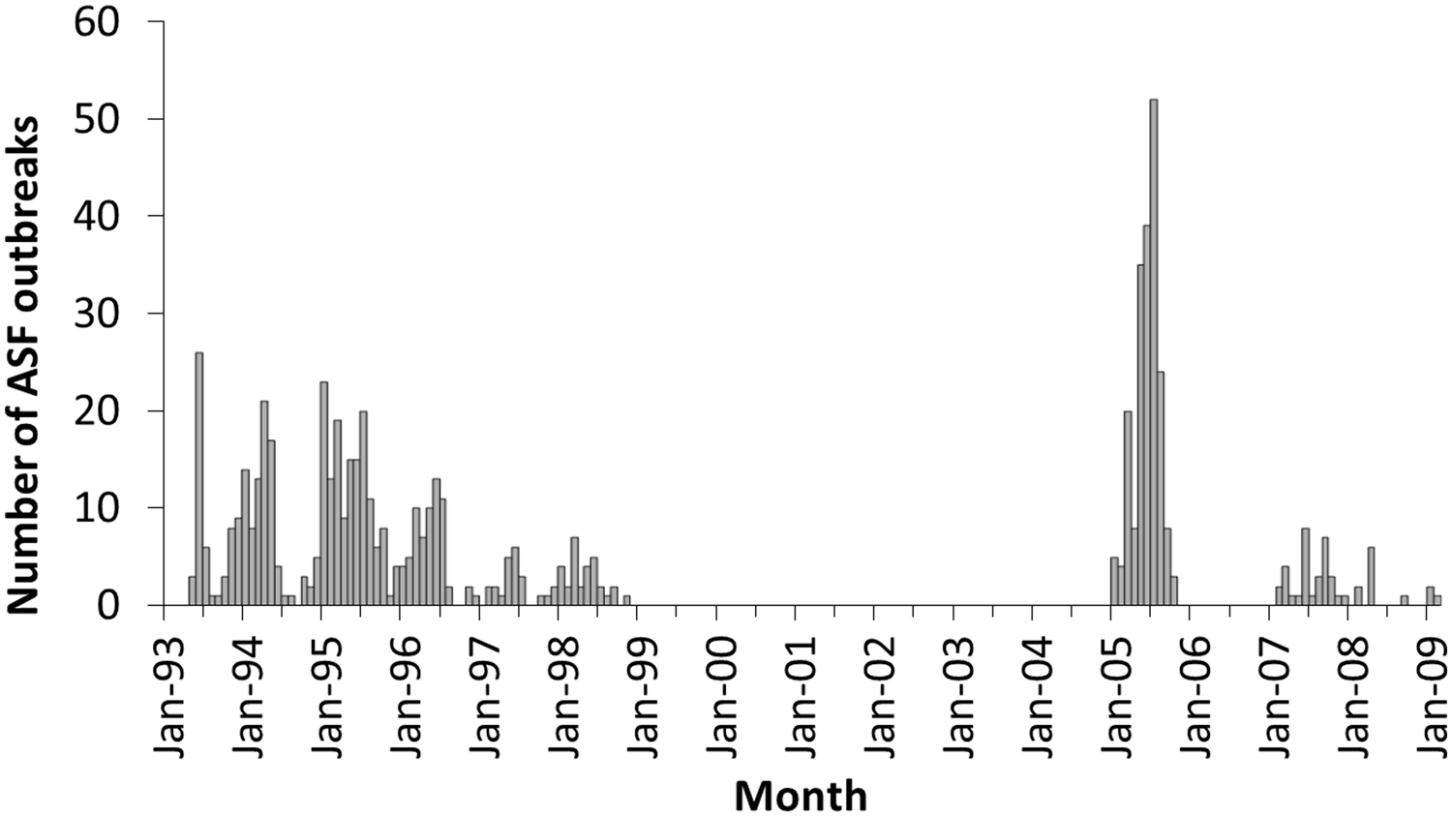
Epidemic curve of the ASF notified outbreaks in Sardinia from 1993 to 2009

African swine fever outbreaks in Sardinia were spatially clustered, as indicated by the two significant clusters found with the Bernoulli scan statistic model (**Figure 2**) and by the much larger value of the DIC when the spatially structured random effect *Si* was removed from the model (DIC = 408.96), compared with the model with *Si* implemented on it (DIC = 267.00). The monthly number of ASF reported outbreaks from 1993 and 2009 is presented in **Figure 3**.

**FIGURE 4:**
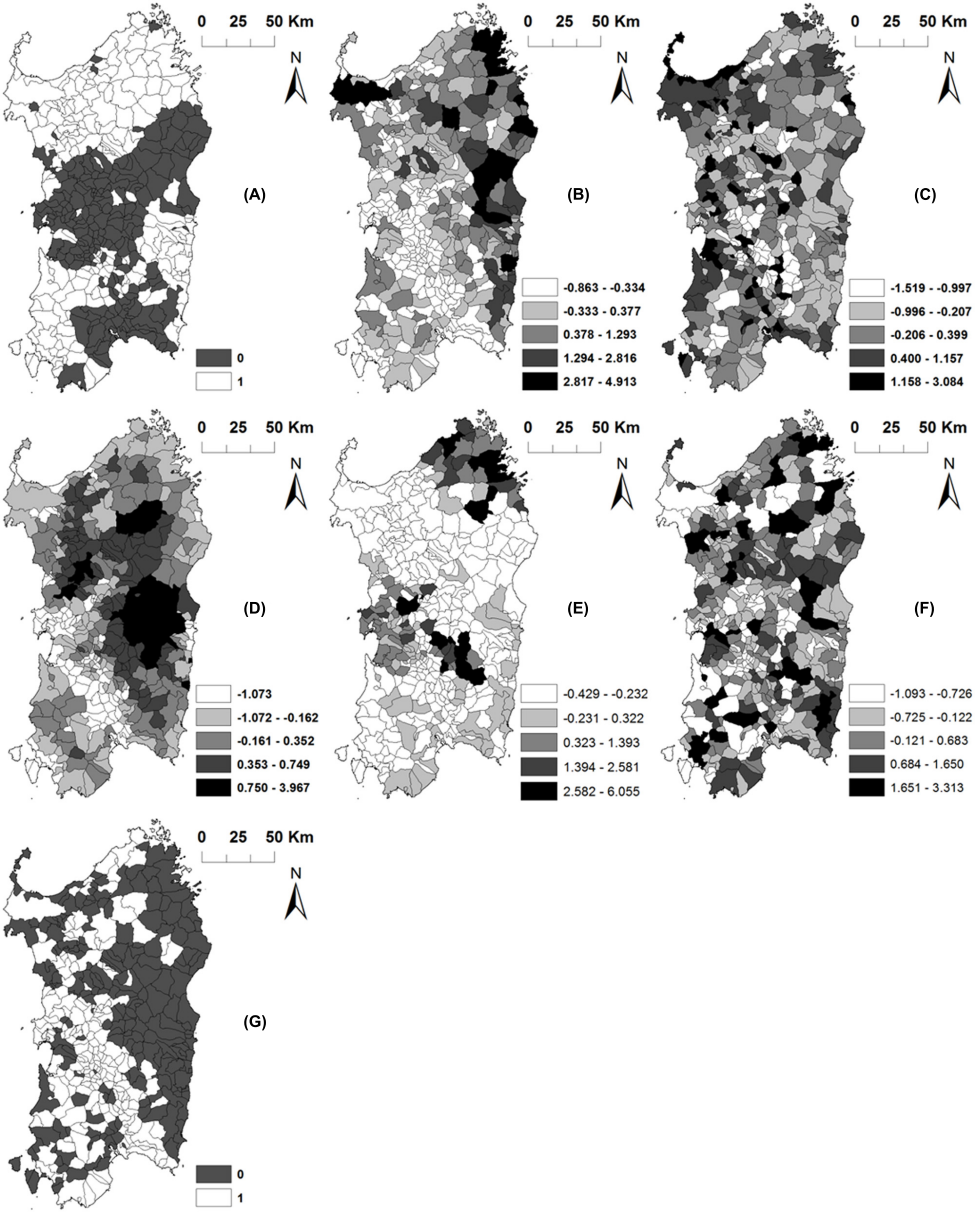
Spatial distribution of the significant variables included in the final model. Maps represents: **(A)** the proportion of open farms with at least one census, **(B)** the number of confined (closed) farms, **(C)** the density of roads, **(D)** the mean altitude, **(E)** the number of open fattening farms, **(F)** the total number of pigs and **(G)** the proportion of open farms. Jenks natural breaks classification method was used for categorization of quantitative variables **(B–F)**.

The model that best fitted the data (DIC = 220.88) included 14 variables; however, only four of them (namely, the proportion of open farms with at least one census, the number of confined/closed farms, the density of roads and, the mean altitude) were significantly (*P* < 0.05) associated with ASF status (**Table 2**). The number of open fattening farms, the total number of pigs, and the proportion of open farms were marginal significant (*P* < 0.1).The spatial distribution of significant variables is shown in **Figure 4**.

**Table 2:** Beta coefficients and Odds ratios for the best fitting model.

Variable	Beta		OR	
	Median	95% PI	Median	95% PI
The proportion of open farms with at least one census (B)	-0.84^i,ii^	[-1.63, -0.08]	0.43^i,ii^	[0.20, 0.93]
The number of confined/closed farms (S)	0.79^i,ii^	[0.21, 1.42]	2.21^i,ii^	[1.23, 4.13]
Open farm density (number of farms/ Km^2^) (B)	-0.61	[-1.41, 0.15]	0.55	[0.24, 1.16]
The density of roads (S)	0.52^i,ii^	[0.03, 1.05]	1.69^i,ii^	[1.03, 2.84]
The mean altitude (S)	0.70^i,ii^	[0.15, 1.27]	2.01^i,ii^	[1.16, 3.55]
The proportion of areas suitable for wild boar presence (B)	-0.25	[-1.09, 0.55]	0.78	[0.34, 1.73]
The number of open fattening farms (S)	0.40^ii^	[-0.07, 0.90]	1.49^ii^	[0.93, 2.47]
The total number of pigs (S)	0.45^ii^	[-0.10, 1.03]	1.56^ii^	[0.91, 2.80]
The number of open farms for self-consumption (S)	-0.09	[-0.60, 0.43]	0.91	[0.55, 1.54]
The number of farms with incoming or outgoing pig movements (B)	-0.29	[-1.05, 0.45]	0.75	[0.35, 1.58]
The total number of open farms (B)	-0.36	[-1.17, 0.41]	0.69	[0.31, 1.51]
Proportion of open farms (number of open farms/total farms; B)	-0.69^ii^	[-1.50, 0.08]	0.50^ii^	[0.22, 1.08]
The mean farm size (number of pigs/number of farms; B)	-0.38	[-1.18, 0.42]	0.69	[0.31, 1.52]
Pig density (number of pigs/Km^2^; B)	-0.31	[-1.09, 0.47]	0.74	[0.34, 1.60]
The total number of pigs (S) × The density of roads (S)	0.29	[-0.18, 0.78]	1.33	[0.84, 2.19]
The total number of pigs (S) × The mean altitude (S)	0.06	[-0.37, 0.52]	1.06	[0.69, 1.68]

*B, Binomial variable (codification 0/1 using median); S, Standardized variable*.

*^i^Significant coefficients of the best fitting model using the 95% prediction interval and ^ii^the 90% prediction interval*.

**FIGURE 5:**
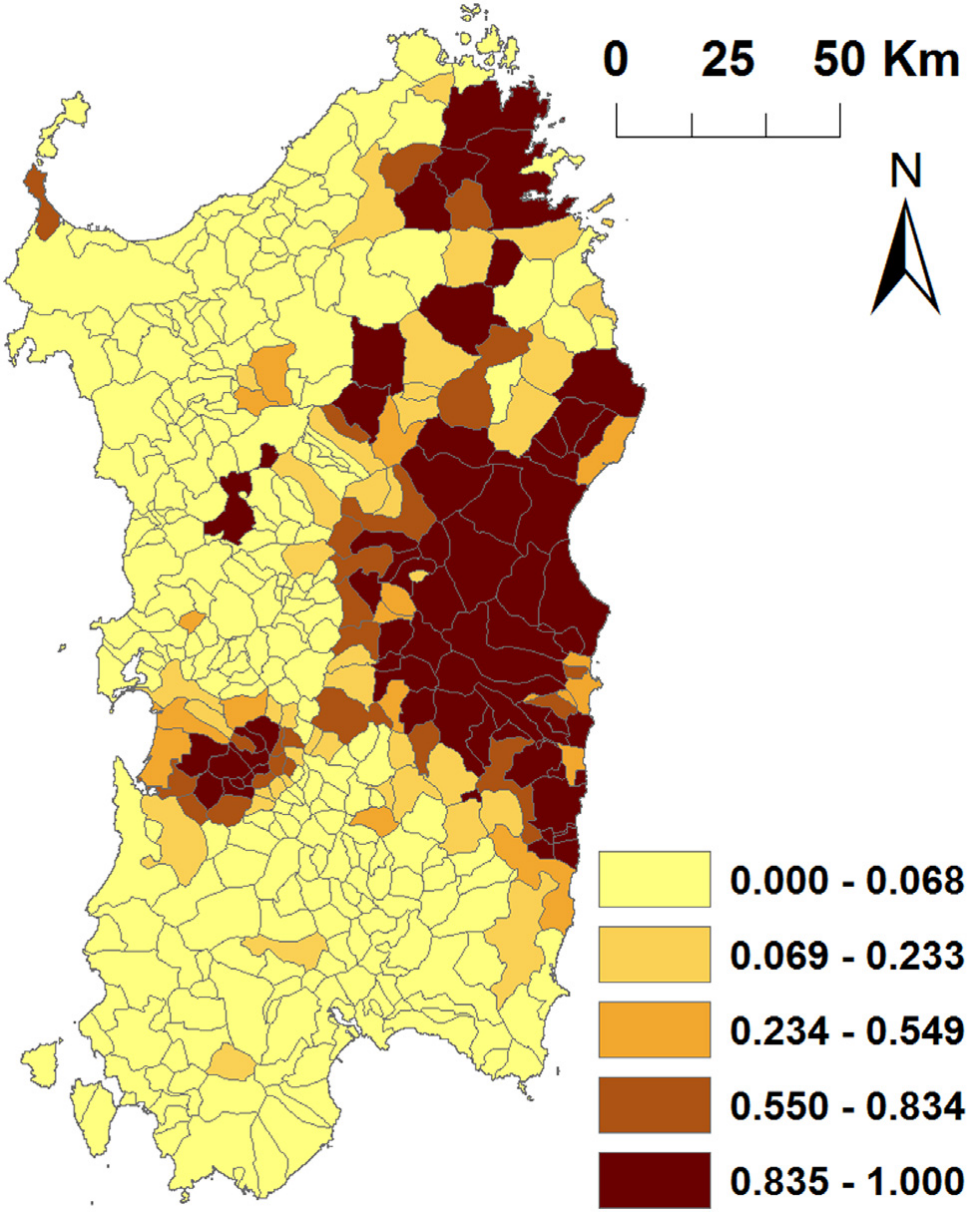
Posterior (predicted) probability of ASF outbreaks in Sardinia obtained with the final model. Jenks natural breaks classification method was used for selection of the cut-offs of the categories.

The posterior probability of ASFV occurrence in Sardinia predicted by the fitted model resembled the spatial structure of the disease observed in the data, with areas at highest risk concentrated in Nuoro, Ogliastra, Olbia-Tempio, and Oristano provinces (**Figure 5**).

**FIGURE 6:**
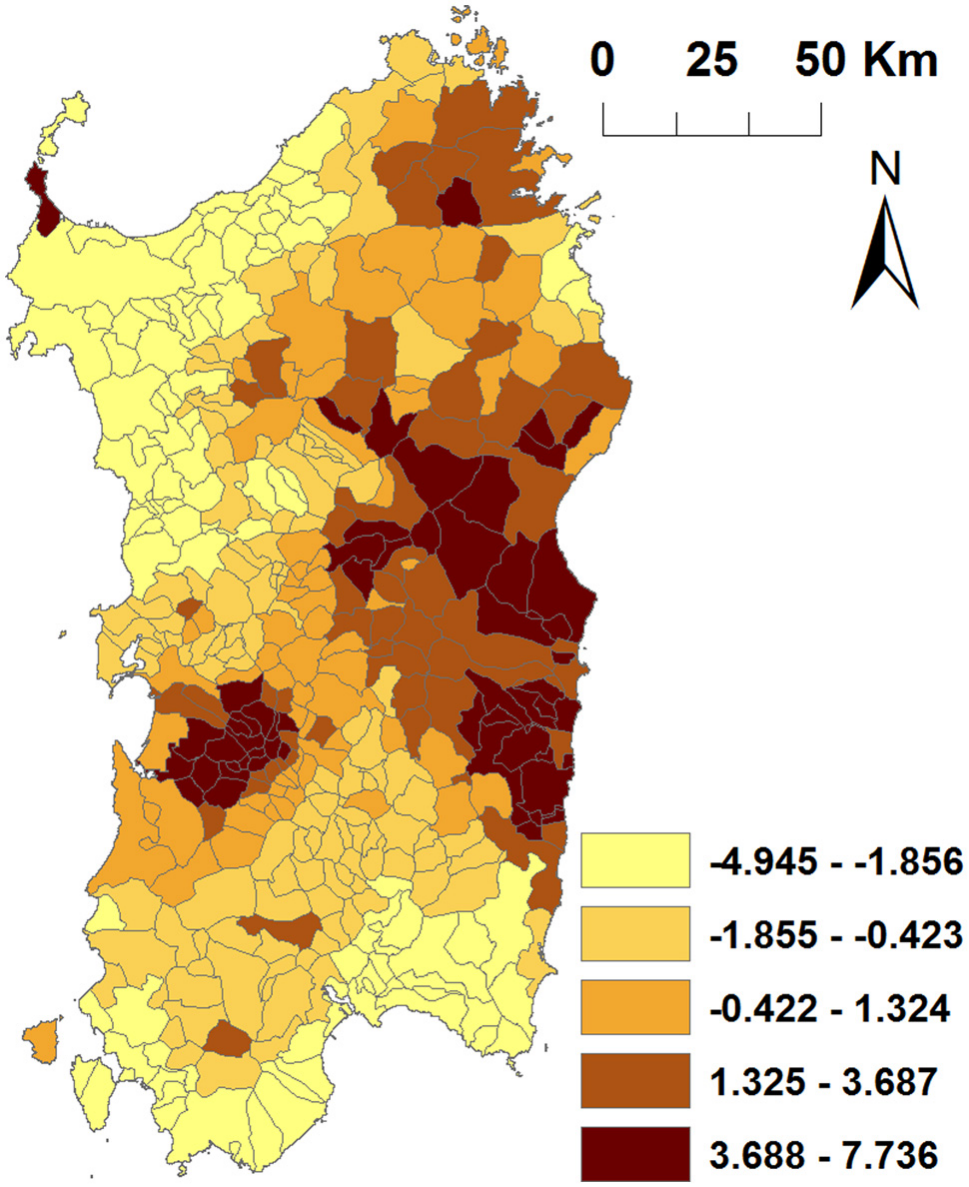
Spatially structured random effects included in the final model for ASF in Sardinia. Jenks natural breaks classification method was used for selection of the cut-offs of the categories.

The SD of *S_i_* (3.497) was substantially larger than SD of *U*_i_ (4.77⋅10^-6^) in the final model (**Figure 6**), which indicates that effects not included into the model were spatially structured.

Two interactions were also found to contribute to the ASF prediction in the final model (**Table 2**). Specifically, in communes where the total number of pigs was greater than the median, an increase in the density of roads was associated with an increase in the predicted probability of ASFV occurrence in Sardinia. Similarly, communes where the total number of pigs was smaller than the median of all communes, an increase in the mean altitude was associated with an increase in the predicted probability of ASFV.

Convergence of the model was obtained after the first 200 iterations and no problems with autocorrelation were identified in the autocorrelation plots for the posterior inferences (data not shown).

## Discussion

Eradication of ASF in Sardinia seems to be far of being accomplished. From 1993 to 1998, the number of ASF outbreaks, which were mainly concentrated in the province of Nuoro, started to decrease, reaching minimum levels in 1999 but, in 2005 a large number of outbreaks were declared in Oristano province and in other areas outside the historical risk area of Nuoro, which emphasized the need to improve eradication efforts. The study presented here was aimed to better understand the nature and extent of epidemiological risk factors for ASFV occurrence in Sardinia in the last 15 years, after the beginning of the eradication plan in 1993.

An unexpected result of the model indicated that the number of confined (closed) farms was associated with high risk of occurrence of ASFV. This relation may be either the effect of a high detection and/or notification of outbreaks in those types of farms, or the consequence of risky management practices, particularly in small-size back yard closed farms, such us the use of waste containing pork products to feed pigs. These results may be in agreement with the hypothesis presented in previous studies by Mannelli et al. (1997), suggesting that the use of garbage to feed pigs was the main way of ASF infection in small farms in Sardinia. It should be noticed that most of the confined farms in Sardinia are small size with poor or no biosecurity measures that favors the access of pigs to contaminated waste food or the contact between infected and susceptible individuals. Only a limited number of those confined farms are large size and have an intensive type of production and high biosecurity level.

The number of open fattening farms was also considered to be a risk factor (OR = 1.49) for the occurrence of ASFV. This may be associated to a greater stocking density, higher/more frequent introduction of pigs on farm or other husbandry practices related to this production system. However, this result may also reflect one of the hypotheses suggested by regional authorities (Sardegna Salute, 2011) regarding the intentional introduction of the disease into some areas. The system of farmer’s compensation after an ASF-outbreak in Italy, which is regulated by the Law n.218/88 (available for example at: http://www.codima.info/trunk/nor_file_39_l218-88.pdf), essentially provides to the breeder the 100% of the value of slaughtered animals, calculated on the basis of the pig market of Modena (Borsa Merci di Modena, 2011). The value of the slaughtered animals, considered reasonable in relation to animals kept in industrial breeding farms, in some cases may be excessive for Sardinian pigs, bred using non-professional type of farming, and mainly when referring to fattening pigs. In such cases, the possibility to receive an extra-profit from the compensation has been suggested to be an incentive to intentionally introduce the disease in their herds for speculative purposes.

It is also noteworthy that communes in which the proportion of areas suitable for wild boar presence, the number of open farms for self-consumption, the number of farms with incoming or outgoing pig movements, the herd size, or the pig density was above the median of all communes were at low risk (although not significant) for ASFV, as indicated by ORs values <1 (**Table 2**). These results suggest, that the role that wild boars play in causing ASF outbreaks is not crucial in Sardinia, which is in agreement with previous studies (Laddomada et al., 1994; Mannelli et al., 1997, 1998). Actually, it has been suggested that wild boars are often infected from domestic pigs in open grazing areas. This hypothesis is supported by the progressive disappearance of classical swine fever and ASF in wild boar only by controlling the grazing of domestic pigs, and by the time sequence of the reports of disease, which occur first in domestic pigs and after in wild boars. However, the potential role of wild boars as reservoir of the disease during short time periods is largely unknown and it has been suggested to be conditioned by several factors such as population dynamics, hunting pressure, and climatic factors such as fires and drought (Sardegna Salute, 2011). Therefore, wild boards demographics should not be neglected in the design of an effective eradication program in areas where extensive farming is practiced.

The association between the total number of pigs, the density of roads and the ASFV occurrence per commune may be explained, at least in part, by a higher risk of infection thought vehicles or other transport-associated fomites in those areas. Other possible explanation may be the potential illegal trade of pigs, in agreement with some speculations (Sardegna Salute, 2011). Similarly, the association between ASFV occurrence, small number of pigs and high altitude per commune may have three alternative explanations. One potential explanation is that there is a longer persistence of the virus in the environment in those mountainous areas. A second one may be that not easily accessible areas located at high altitudes have low biosecurity measures and less control by veterinary authorities, which increases the risk for ASFV infection. Alternatively, another explanation may be that those remote areas are more likely to introduce illegal pigs (not declared/not censed). In any case, it is interesting to note that those remote areas with low number of pigs are at higher risk for ASF than other areas of the Island. This result highlights the importance of increasing efforts not only to control the population of pigs (reducing the illegal pigs) but also to regulate the contact among pig populations in those territories. In this regard, it could be very important to increase the surveillance in the “*terre pubbliche*” or “*pascoli comunali*,” which are open areas that farmers can freely use to allocate and feed their animals (including pigs), and which may promote the ASFV transmission. Those open areas not only have low biosecurity and are difficult to disinfect and control, in case of ASF infection, but also are places where pigs may easily contact with swill feeding or waste coming from picnics and other celebrations there.

Conversely, the proportion of open farms with at least one census in the commune was the most significant protective factor (OR = 0.43). Notice that censed farms are those in which the number of pigs on farm has been provided either by the farmer or by the veterinary authority. Consequently, communes with most of their farms censed (i.e., controlled by the veterinary authority) are less likely to have ASF outbreaks than not censed (i.e., unsupervised) farms. This relation may suggest that communes with a large proportion of not censed farms are more likely to have illegal pigs and/or risky management practices, and, in consequence, more ASF outbreaks than communes with a large proportion of censed farms. This result highlights the importance that relatively simple measures, such as a compulsory registration of the pig population, may have in the control ASF in Sardinia. Results suggest that such measure may, for example, help to reduce the number of farms holding illegal pigs, and consequently, risky farm practices that promote ASF spread.

Counter-intuitively, farms for self-consumption as well as areas with high pig density and large herd sizes and intense trade/movement of animals, seem to have less risk of ASFV occurrence than the background risk of the region. This finding may reflect that those types of farms have less contact with infected domestic pigs or pig products as a result of less trade, in the case of farms for self-consumption, or higher biosecurity measures, in the case of large/industrial pig farms.

In addition, unmeasured risk factors are likely associated with ASF in Sardinia as it has been suggested by the *S* and *U* components of the model. Spatial structure random effect (S) was considerably higher than unstructured effect (*U*). It means that these unmeasured factors are specifically spatial located, mainly in east part of the Island, and could be related, at least in part, with characteristic cultural and local pig husbandry management associated to specific areas. These factors could be associated with the illegal presence of pigs or pig trade, or the use of waste containing raw pork to feed of pig, which are factors difficult to estimate and for which not much information is available.

Unfortunately, information on the value of covariates prior to 2009 was not available to us and for that reason, an assumption of this study is that values recorded in 2009 are representative of the entire period. Also, certain data such as the number of pigs or number of pig farms were aggregated to the commune level and that some variables such as roads or water areas were polylines and had also to be transformed to the commune level. In general, aggregating data may lead to potential ecological bias. This study used the smallest unit of analysis to reduce this potential ecological bias and to facilitate the decision making process in the control and eradication of the disease. In this regard, commune was assumed to be the best level of aggregation as it is relatively small (median = 64 km^2^) and it is the unit of analysis and policy making used in the ASF eradication plan (Decreto assessore igiene sanita’ 6 luglio 2010 n. 33/1302, 2011).

## Conclusion

Future eradication programs should reinforce the compulsory registration of the pig population in Sardinia and the surveillance and control measures in those “*terre pubbliche*” or “*pascoli comunali*” used by pigs. Those measures will help to reduce the number and trade of illegal pigs and to minimize the ASFV transmission in the Island. Results presented here suggest that it is fundamental to adapt the ASF preventive and control strategies considering all risk factors as well as the socio-cultural, productive and economical conditions of the region, in order to eradicate ASF from Sardinia and to achieve the ultimately goal of eradicating the disease from the EU and other regions of the world.

## Author Contributions

BL collected and processed the data from Anagrafe Nazionale Zootecnica – Statistiche, performed analyses and drafted the manuscript. AP provided assistance on the model development, supervised the analysis and gave major advices about the methodology and interpretation of results. FF and SR contributed with some of the data, provided expertise and technical considerations regarding the ASF eradication program in Sardinia and assisted on the interpretation and discussion of results. LM provided assistance on the data collection and processing and together with JS-V assisted on the study design and discussion of results. All authors read, reviewed, and approved the final manuscript.

## Conflict of Interest Statement

The authors declare that the research was conducted in the absence of any commercial or financial relationships that could be construed as a potential conflict of interest.
